# Peroxisome Proliferator–Activated Receptor-α: A Pivotal Regulator of the Gastrointestinal Tract

**DOI:** 10.3389/fmolb.2022.864039

**Published:** 2022-04-26

**Authors:** Yue-Xin Guo, Bo-Ya Wang, Han Gao, Rong-Xuan Hua, Lei Gao, Cheng-Wei He, Ying Wang, Jing-Dong Xu

**Affiliations:** ^1^ Department of Oral Medicine, School of Basic Medical Sciences, Capital Medical University, Beijing, China; ^2^ Eight Program of Clinical Medicine, Peking University Health Science Center, Beijing, China; ^3^ Department of Physiology and Pathophysiology, School of Basic Medical Sciences, Capital Medical University, Beijing, China; ^4^ Clinical Medicine of “5+3” Program, School of Basic Medical Sciences, Capital Medical University, Beijing, China; ^5^ Department of Biomedical Informatics, Faculty of Biomedical Engineering, Capital Medical University, Beijing, China; ^6^ Department of Dermatology, Tongren Hospital, Capital Medical University, Beijing, China

**Keywords:** peroxisome proliferator–activated receptor (PPAR)-α, gastrointestinal diseases, metabolism, transcription, disorder

## Abstract

Peroxisome proliferator–activated receptor (PPAR)-α is a ligand-activated transcription factor distributed in various tissues and cells. It regulates lipid metabolism and plays vital roles in the pathology of the cardiovascular system. However, its roles in the gastrointestinal tract (GIT) are relatively less known. In this review, after summarizing the expression profile of PPAR-α in the GIT, we analyzed its functions in the GIT, including physiological control of the lipid metabolism and pathologic mediation in the progress of inflammation. The mechanism of this regulation could be achieved *via* interactions with gut microbes and further impact the maintenance of body circadian rhythms and the secretion of nitric oxide. These are also targets of PPAR-α and are well-described in this review. In addition, we also highlighted the potential use of PPAR-α in treating GIT diseases and the inadequacy of clinical trials in this field.

## Highlights

In this review, after briefly introducing the characteristics of the PPAR family in the liver and cardiovascular system, we highlighted the specialties of PPAR-α and summarized its role in the gastrointestinal tract. It is responsible for the regulation of nutrient uptake and mediation of the inflammatory process. Moreover, studies also reported its participation in the maintenance of gastrointestinal circadian rhythms or circadian clock and satiety. These may provide novel and therapeutic targets for the treatment of gastrointestinal and systemic diseases.

## Introduction

Since the discovery and cloning by Issemann et al. in 1990, peroxisome proliferator–activated receptors (PPARs) have received increasing attention for their multiple functions (Issemann and Green, 1990). Three subtype proteins found within the family are known as PPAR-α, PPAR-γ, and PPAR-β/δ, regulating the lipid metabolism and inflammation state (Dreyer et al., 1992; Bordet et al., 2006). They share common functions in metabolism and inflammatory regulation but are distinct from one another in both the distribution patterns and target molecules (Braissant et al., 1995). The common structure of the ligand-binding domain (LBD) in the shape of the letter Y laid the basis for the similarity and differences among this protein family (Mandard et al., 2004). The first arm containing hydrophilic amino acid residues is responsible for ligand binding and exists in all three subtypes, while the remaining two parts consisting of far fewer amino acid hydrophilic residues account for the specialties among them. PPAR-α is a transcription factor belonging to the nuclear receptor superfamily and could be activated by fibrates, eicosanoids, and fatty acids (Forman et al., 1997). However, contrary to steroid hormone receptors acting as homodimers, transcriptional regulation by PPARs requires heterodimerization with the retinoid X receptor (RXR; NR2B) in the same receptor superfamily (Mandard et al., 2004).

PPARs are ligand-activated transcription factors originally known to be activated by hepatocarcinogens and lead to peroxisome proliferation (Desvergne and Wahli, 1999). They are detected in a wide range of tissues, including endothelial and muscular cells and macrophages (Mφs) and monocytes. This endows them with a wide range of roles, including immune functions all over the body and regulations of a specific organ (Mandard et al., 2004). The well-recognized roles in alleviating heart dysfunction and hypertension in the cardiovascular system have been well-characterized (Goikoetxea et al., 2004; Usuda and Kanda, 2014), and their abilities to regulate fatty acid transportation and oxidation in the liver have been well-illustrated, further unveiling its association with various kinds of liver injuries (Botta et al., 2018; Kong et al., 2021). These could lead to some systematic diseases including diabetes and result in pathological dysfunctions in multiple organs (R. Moschen et al., 2012). In the meantime, studies have verified their roles in peripheral and neural inflammation (Piomelli, 2013). However, although much effort has been put into investigating its roles in the cardiovascular system, investigations on its roles in the gastrointestinal tract were relatively less. Recent analysis has certificated the distribution and activation of PPAR-α in the GIT with a higher level in the more differentiated cells near the lumen compared to those residing in the crypts (Mansén et al., 1996). Furthermore, studies also confirm the expression of PPAR-α in enterocytes along the small intestine with the highest levels in the duodenum and the jejunum. A higher level of PPAR-α is also found in villus tips than in crypts (Bünger et al., 2007). This expression pattern is similar to that of several other genes involved in dietary fat absorption, including microsomal triglyceride transfer protein (Mttp), diacylglycerol acyltransferase 1 (Dgat1), fatty acid translocase (Cd36), and fatty acid transport protein 4 (Fatp4), and lay the foundation for their wide interactions (Suzuki et al., 2009).

In this review, the roles of PPAR-α in the development of inflammation and regulation of metabolism are depicted and show its broad regulatory effects in the GIT and the whole body. Meanwhile, as agonists and antagonists are commonly used as drugs for the cardiovascular system (CVS), we evaluated the possibilities of their use in treating GIT diseases.

## A Pivotal Regulator of Metabolism

As mentioned earlier, PPAR-α is involved in regulating the expression of various genes in lipid metabolism. However, despite the well-depicted regulation of genes associated with lipid metabolism in the liver, the regulation of genes by PPAR-α in the intestine is relatively less described (Steineger et al., 1994). In fact, in addition to the similarity in expression modes, Affymetrix arrays and quantitative RT-PCR analysis have demonstrated a PPAR-α–dependent upregulation of eight genes concerning transporters and phase I/II metabolism during fasting (the details of these genes are shown in Table 1) (van den Bosch et al., 2007). Several other studies also corroborated the increase of PPAR-α in mice and the downregulation of genes related to lipid metabolism (Escher et al., 2001; Shimakura et al., 2006).

**TABLE 1:** Metabolic genes regulated by PPAR-α.

Abbreviation	Full name	Localization	Reference
Cypt4a10	Cytochrome P450, family 4, subfamily a, polypeptide 10	Microsome	Wu et al. (2020)
Abca1	ATP-binding cassette, sub-family A (ABC1), member 1	Nucleoplasm and vesicles	Sasaki et al. (2019)
Smct1 (Slc5a8)	Solute carrier family 5 (iodide transporter), member 8	Apical	Sivaprakasam et al. (2017)
Sert (Slc6a4)	Solute carrier family 6 (neurotransmitter transporter, serotonin), member 4	Basolateral	Mastinu et al. (2012)
Dtd (Slc26a2)	Solute carrier family 26 (sulfate transporter), member 2	Apical	Haila et al. (2001)
Slc25a36	Solute carrier family 25, member 36	Mitochondria	Lee et al. (2018)
Chst4	Carbohydrate (chondroitin 6/keratan) sulfotransferase 4	Intracellular membrane	Yu et al. (2018)
Mgst1	Microsomal glutathione S-transferase 1	Intracellular membrane	Cui et al. (2010)

Intestinal fatty acid–binding proteins (IFABPs) are important for regulating the uptake and transportation of the long-chain fatty acids (LCFAs) and significant biomarkers of gastrointestinal diseases (Holehouse et al., 1998; Kokesova et al., 2019). Detected more in proximal than in distal small intestine (Poirier et al., 1996), the IFABP expression in the rat jejunum showed significant enhancement during the postnatal development, concomitant with the increased mRNA level of PPAR *in situ*. Electrophoretic mobility shift assays revealed the existence of the PPAR-α-9-cis-retinoic acid receptor (RXRα), a heterodimer whose binding activities could be enhanced by an additional PPAR-α agonist WY-14643 (Mochizuki et al., 2001). Although this is inconsistent with some previous findings that the levels of PPAR-α and IFABPs show contrary variations under some treatment (Poirier et al., 1997), the regulation of metabolism by PPAR-α *via* gene transcription might be undeniable as more investigations utilizing different types of the PPAR-α agonist witnessed a concomitant increase of the IFABP level with PPAR-α (Mallordy et al., 1995).

Meanwhile, studies comparing the expression mode of genes between obesity-resistant A/J and obesity-prone C57BL/6J mice show a prominent upregulation of genes regulating lipid metabolism. However, this increase is restricted in the small intestine with no significant change in other organs such as the liver and white adipose tissue. Experiments in mouse Caco-2/TC7 cells and in human jejunal biopsies show that PPAR-α activation using WY-14643 increases the expression of ATP-binding cassette transporter A1 (ABCA1) (Knight et al., 2003). However, when WY-1463 was replaced by fibrates, the levels of both ABCA1 and protein-1c gene (SREBP-1c) increased. This is concomitant with the increase in the expression of genes modifying cholesterol trafficking and the decreased capacity of cholesterol esterification. Meanwhile, other findings show that the usage of fenofibrate, a selective PPAR-α agonist, and elafibranor (GFT505), a selective PPAR-α/δ agonist, did not remain the same in different experiments (Colin et al., 2013). Similar experiments further verified that this modulation could also be applied to the expression of genes regulating lipid metabolism (Kondo et al., 2006). For example, intraperitoneal (IP) administration of pirinixic acid (Wy-14643), a selective and highly potent PPAR-α agonist, stimulates fatty acid oxidation (FAO) and ketogenesis in the intestine This is concomitant with a significant increase in the expression of cytochrome P450 1A1(CPT 1A1) in the jejunum and duodenum and of HMG-CoAS2 in the jejunum (Stavinoha et al., 2004; Sérée et al., 2004). However, in this experiment neither CPT 1A nor HMG-CoAS2 expression was increased in the liver, suggesting a pivotal role the intestine plays in this regulation (Karimian Azari et al., 2013). Other genes in this type of regulation include fatty acid translocase (FAT)/cluster of differentiation 36 (CD36), fatty acid transport protein (FATP), NPC1L1, Acox1, Fabp1 (Hutch et al., 2020), and mitochondrial aspartate aminotransferase (mAspAT) (refer to Table 2 for more detailed information) (Uchida et al., 2011; Roberts, 1989; Valasek et al., 2007). Contrarily, Pan et al. found a PPAR-α independent way of OEA to increase the secretion of triacylglycerols, ApoB, and MTP in differentiated Caco-2 cells and primary enterocytes (Pan et al., 2018). Consistently, Mariana et al. also found no pertinence between the component of nutrient transporters and the level of PPAR-α (Losacco et al., 2018). Also, in many experiments, the level alteration in the intestine differs from that in the liver and the range of targeted genes varied with different kinds of agonists, indicating a complex mechanism waiting for investigation (Motojima et al., 1998). The use of PPAR-α agonists, both natural and synthetic, is an effective and widely accepted method to examine its functions (as shown in Table 2) (Lefebvre et al., 2006). Further analysis of these molecules, including oleoylethanolamide (OEA), palmitoylethanolamine (PEA), and WY-14643, provides the foundation for the understanding of the broad variety of PPAR-α functions.

**TABLE 2:** Genes regulating fat metabolism by PPAR-α.

Abbreviation	Full name	Functions	Reference
FATP	Fatty acid transport protein	Transport fatty acids	Ochiai et al. (2019)
FAT/CD36	Fatty acid translocase	Fatty acid translocase	Haidari et al. (2021)
NPC1L1	NPC1-like intracellular cholesterol transporter 1	Membrane transportation	Long et al. (2021)
Acox1	Acyl-CoA oxidase 1	Rate-limiting enzyme of the peroxisomal beta-oxidation pathway acyl-CoA oxidase 1	Vluggens et al. (2010)
Fabp1	Fatty acid–binding protein 1	Transport long-chain fatty acids through cell membranes and mediate intracellular transport as a chaperone	Valizadeh et al. (2021)
mAspAT	Mitochondrial aspartate aminotransferase	Mitochondrial aspartate aminotransferase	Ochiai et al. (2019)

**TABLE 3:** Representative agonists of PPAR-α.

Classification	Name	Source	Usage	Limitations	References
Natural and multi-functional acids	Oleoylethanolamide	Oleic acid-derived	Diabetes	Mechanisms not fully clear	Laleh et al. (2019)
	Palmitoylethanolamine	Naturally occurring lipid that falls under the fatty acid amide group	Neuroinflammation	Multi-functions and lack of clinical data	Skaper et al. (2015)
Mimetic acid	WY-14643	A versatile fatty acid mimetic	Cancer and inflammation	Not so typical as a PPAR agonist	Pollinger and Merk (2017)
Novel PPARα-selective agonists	9-hydroxy-10(E),12(E)-octadecadienoic acid	Koji extract	Decreases plasma triglyceride and glucose levels and body weight gain	Selectivity unclear	Yoshizaki et al. (2014)
Novel PPARα/γ dual agonists	LDT477		Treatment of metabolic and inflammatory diseases	*In vivo* effects remain unknown	Maltarollo et al. (2018)

**FIGURE 1:**
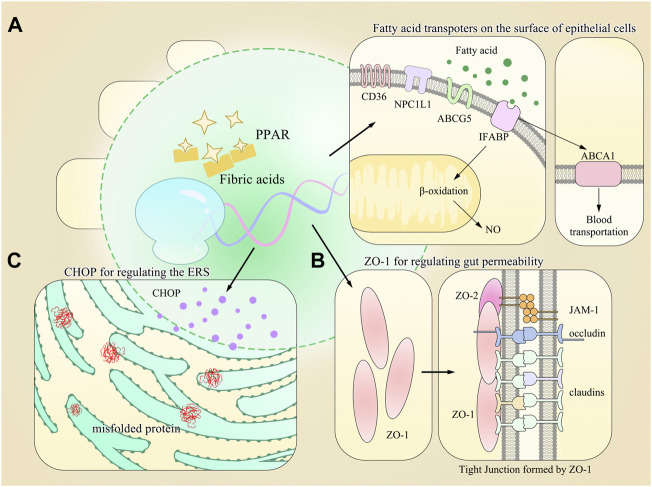
Model diagram of PPAR-α-regulating gene expression and multiple physiological processes in the gut. **(A)** Multiple types of fatty acid transporters are found on the surface of gastrointestinal epithelial cells, and most of their synthesis requires the activation of PPAR-α. **(B)** Other proteins maintaining the homeostasis of GIT are also regulated by PPAR-α, such as ZO-1 for gut permeability. **(C)** PPAR-α also regulates the expression of CHOP, which is responsible for regulating the endoplasmic reticulum stress (ERS).

All these findings show novel roles of PPAR-α in the intestine compared to those in the liver and are worth more investigations for full elucidation (Refer to Figure 1 for visual understanding).

### Oleoylethanolamide, a Widely Accepted Endogenous Peroxisome Proliferator–Activated Receptor-α Agonist Used in Investigations

Oleoylethanolamide (OEA) is a kind of endogenous PPAR-α agonist with high affinity and plays an important role in the treatment of obesity and atherosclerosis. It is a structural analog of the endocannabinoid anandamide, an endogenous free fatty acid known for its role in regulating lipid metabolism (Rodríguez de Fonseca et al., 2001). It could be derived from digestion products by intestinal microbes and could be secreted endogenously by enterocytes (Obici et al., 2002). Meanwhile, it is also synthesized by astrocytes and neurons and could serve as a significant neurotransmitter regulating satiety (Cani et al., 2004). Most of these functions are mediated by PPAR-α, making it a potential target toward diabetes and giving it increasing significance considering the relationship with cardiovascular and neuron dysfunctions mentioned earlier (Koethe et al., 2009). It is also reported to bear a higher affinity compared with the other two agonists (Lo Verme et al., 2005; Brown et al., 2017). Moreover, the use of OEA supplements has been approved by the FDA for the treatment of obesity and shows prospective effects (Brown et al., 2018a). This is contrary to some previous studies revealing the side effects of OEA, indicating the requirement for more detailed studies (Nielsen et al., 2004; Brown et al., 2018b). These regulations in general help with the maintenance of a proper level of PPAR-α in the intestine and the homeostasis under its regulation.

Apart from the roles in regulating metabolite-associated gene expressions, OEA is also found to lower body weight and relieve hyperlipidemia in obese rats *via* the regulation of NO synthesis (Fu et al., 2003). Further analysis showed that it could also regulate satiety through a paracrine PPAR-α–mediated mechanism involving the recruitment of afferent sensory fibers (DiPatrizio and Piomelli, 2015). OEA produced by small-intestinal enterocytes during dietary fat digestion activates PPAR-α to trigger an afferent signal that causes satiety (Igarashi et al., 2017). Using a rat model of Roux-en-Y gastric bypass (RYGB), Hankir et al. found that marked reductions in fat appetite are due to enhanced gut lipid sensing through PPAR-α, which is in turn transmitted to the central nervous system (CNS) by sensory vagal afferents, culminating in increased dorsal striatal D1R signaling (Hankir et al., 2017). However, using multiple dopamine D2/D3 receptor agonists and celiac superior mesenteric ganglionectomy (CGX) or subdiaphragmatic vagal deafferentation (SDA), Shahana et al. showed that IP OEA’s anorectic effect may be secondary to impaired locomotion rather than physiological satiety and that vagal afferents do not mediate exogenous OEA’s anorectic effects. They also suggested a role for spinal afferents in addition to an alternative, non-neuronal signaling route (Fedele et al., 2018). Taken together, these findings raised the possibilities for the treatment of eating disorders by OEA and other PPAR-α–related products (Bünger et al., 2007).

## Important Regulators of Inflammation

Intestinal bowel diseases (IBD), including mainly Crohn’s disease and ulcerative colitis, are relapsing and chronic GIT disorders becoming increasingly prevalent all over the world (Windsor and Kaplan, 2019). Despite the poor understanding of its pathogenesis, tissue examinations of various patients show different levels of mucosa injuries concomitant with the courses of diseases (Ahmad et al., 2017). These findings suggest the dysregulation of epithelial cell functions resulting from stimulations both directly from the lumen contents and cytokines secreted by lymphocytes and Mφ. As is known that metabolites could serve as mediators of IBD, it is reasonable to understand the underlying indirect role PPAR family proteins plays in the process of IBD (Roediger and Nance, 1986). Meanwhile, transcriptomic and proteomic profiling of human colon biopsy specimens showed the downregulation of PPAR signaling pathways in IBD (Jin et al., 2019). Studies also found disruption of the protective roles of PPAR-α agonists in PPAR-α-KO mice, indicating the pivotal functions it may have in the course of GIT diseases (Capasso et al., 2014). Also, the level of PPAR-α is decreased in a resection model of short bowel syndrome and is consistent with its level alteration in high malignant human tissue mucosa (Wang et al., 2007). Using human HCA7 cells, Jackson et al. further convinced the activation of PPRE-tk-luc, a PPRE-driven reporter gene, by PPAR-α using the transfection method (Jackson et al., 2003). In the dextran sodium sulfate (DSS)–induced mouse ulcerative colitis model, Manoharan et al. found that PPAR-α regulates the expression of IL-22 and antimicrobial peptides RegIIIb, RegIIIg, and calprotectin (Manoharan et al., 2016). IL-22 is an important member of the IL-10 cytokine family and has bidirectional functions for both anti-inflammation and pro-inflammation (Wei et al., 2020). However, the detailed mechanism by which PPAR-α activated NKp46^+^ ILC3 cells, the major producers of IL-22 under homeostatic conditions in the gut, still remains to be elucidated. Studies also corroborated that PPAR-α played defensive roles in the progression of IBD and CAC mainly *via* the stimulation of antimicrobial peptides RegIIIb and RegIIIg (Zheng et al., 2008; Killig et al., 2014) In interleukin 10 knockout (IL-10^−/−^) colitis mice, treatment with fenofibrate repressed interferon-gamma and IL-17 expression in isolated T cells. Considering the activation of PPAR-α by fenofibrate, this protection could be attributed to PPAR-α and put into clinical uses (Lee et al., 2007). Increased levels of Th17 and Th1 cells in this model may also account for injuries in the GIT as the secretion of IL-17 by Th17 is a core step in the progression of GIT disorders (Yang et al., 2017). An increasing number of other pro-inflammatory factors including IL-1b, IL-6, and TNF-α could be possible reasons for this enhancement in Th17 and Th1 cells. Concomitant with this, DNBS-treated PPAR-α–knockout (PPAR-αKO) mice experienced severer colon injuries accompanied with upregulation of ICAM-1 (Cuzzocrea et al., 2004). The levels of TNF-α and interleukin-1β (IL-1β) were also increased, resulting in antibody-mediated membrane dysfunctions (Stack et al., 1997). The decreased level of ICAM-1 and other adhesion molecules including VCAM-1 and P-selectin reduces the infiltration of neutrophils and ROS formation and thus aggravates the intestinal inflammation (Cuzzocrea et al., 2001). Apart from the anti-inflammatory roles of PPAR-α on DNBS-induced colitis, the functions of PPAR-α could also be enhanced by glucocorticoids (GCs). Other studies also show a less degree of colitis in WT mice compared to that in PPAR-αKO mice with an inhibition of p65 phosphorylation, which is an important regulator of the NF-κB pathway (Riccardi et al., 2009). Similarly, in human enterocytes (Caco-2), Shinsuke et al. also found the involvement of NF-κB after OEA injection (Otagiri et al., 2020). Also, in the splanchnic artery occlusion (SAO) shock model, administration of PEA 5 min before reperfusion significantly reduced the inflammatory parameters, including IL-1β and TNF-α. These effects were at least partly dependent on PPAR-α as the decrease of inflammatory markers was less significant in PPAR-α^−/−^ mice than that in WT ones (Di Paola et al., 2012). In conclusion, all these studies provide novel insights into the roles of PPAR-α in mediating GIT inflammation and provide a potential target for pharmaceutical synthesis.

As an analogy of OEA, palmitoylethanolamide, a well-recognized PPAR-α agonist, could also exert an antiproliferative effect and downregulate VEGF signaling in Caco-2 through selective and PPAR-α-dependent inhibition of the Akt/mTOR pathway (Sarnelli et al., 2016). Several studies have corroborated the effect of palmitoylethanolamide in attenuating the GIT injuries using different models of both humans and mice (Borrelli et al., 2015). Mustafa et al. demonstrated its roles in modulating intestinal permeability in a PPAR-α–dependent method using the antagonist GW6471 (Karwad et al., 2017). In the intestine, PEA treatment also improves all macroscopic signs of UC and decreases the expression of the pro-inflammatory biomarkers, including PGE2, IL-1β, and TNFα. Further analysis shows that this effect is mediated mainly by selective targeting of the S100B/TLR4 axis on ECG and downstream inhibition of NF-кB-dependent inflammation (Esposito et al., 2014). Using mice with chronic intestinal inflammation induced by croton oil, Raffaele et al. found significantly decreased levels of PEA in inflammatory mice which could probably contribute to the exaggerated transition (Capasso et al., 2001). However, Cluny et al. showed no difference in gut mobility between PPAR-αKO and WT mice, indicating a PPAR-α–independent pathway in remaining elucidation (Cluny et al., 2009).

Apart from the roles as a significant mediator in IBD, PPAR-α can also regulate the progress of GIT cancer. Studies have found gastric gavage of the PPAR-α ligand bezafibrate inhibited the DSS-induced colitis by and lowered trefoil factor-2 content in colonic mucosa (Tanaka et al., 2001). It also inhibits the formation of aberrant crypt foci (ACF), which is recognized as a precursor lesion in colorectal cancer (Shivapurkar et al., 1997). Further investigations show increased expressions of cyclooxygenase-2 (COX-2), an important mediator in the development of colonic carcinoma in the human colorectal epithelial cell line HT-29 (Prescott and White, 1996; Ma et al., 2018). This could be explained by previous findings that show COX-2–mediated regulation is one of the important downstream pathways induced by TFF2 and enhanced the COX-2 expression *via* PPAR ligands in some human colorectal epithelial cells (Ikawa et al., 2001; Meade et al., 1999).

## Mediation Between Metabolism and Inflammation

Acknowledged as the “second brain” in the human body, gut microbiota play essential roles in the gastrointestinal tract that is regarded as the largest digestive as well as immune organ (Ridaura and Belkaid, 2015). Based on our aforementioned analysis, PPAR-α exerts a significant influence on the components of gastrointestinal microbes and the host physical health condition *via* the regulation of gene transcription (Rooks and Garrett, 2016; Ashrafian et al., 2019; Hasan et al., 2019). *Lactobacillus* species are significant protectors of the GIT, and the reduction of their number is an important characterization in many GIT diseases (Tan et al., 2020). This regulation is partly mediated *via* the PPAR-α as sub-chronic OEA administration to mice fed with a normal chow pellet diet changes the fecal microbiota profile and shifts the Firmicutes: Bacteroidetes ratio in favor of Bacteroidetes (in particular *Bacteroides* genus). It also decreases the number of Firmicutes (*Lactobacillus*) and reduces the intestinal cytokine expression by immune cells isolated from Peyer’s patches. (Di Paola et al., 2018; Cai et al., 2020; Kersten et al., 1999), Meanwhile, the introduction of probiotic *Lactobacillus plantarum* into simian immunodeficiency virus (SIV)–inflamed intestinal lumen resulted in a higher level of PPAR-α concomitant with a recovered expression of PPAR-α–targeted genes (Crakes et al., 2019). Studies also found that mice fed with high-fat chow and supplemented with the probiotic bacteria *Lactobacillus paracasei* ssp. *paracasei* F19 (F19) exhibit significantly less body fat. This is also accompanied by a higher level of angiopoietin-like 4 (ANGPTL4), a circulating lipoprotein lipase (LPL) inhibitor regulated by PPAR-α and shows the protective roles of it (Aronsson et al., 2010). These findings corroborated the roles of PPAR-α with *Lactobacillus* and provided novel prospects for future studies. Apart from the roles in modulating GIT functions, the effect of this interaction could also alter the physiological and pathological conditions of other organs *via* metabolites transporting in blood as experiments found that exposure to high-fat diets and food deprivation enhances PPAR-α-dependent signaling in the liver and intestine. *Lactobacillus plantarum* FRT10 could also alleviate the high-fat diet-induced obesity in mice *via* regulating the PPAR-α signal pathway (Duparc et al., 2017).

Apart from the roles in regulating metabolism and inflammation, respectively, interactions between PPAR-α and gut microbiota also help with the maintenance of the circadian rhythms. This could be confirmed by its disruption under microbiota depletion and result in activation of the c-Jun expression, leading to the dysregulation of a serious set of genes related to inflammation (Mukherji et al., 2013).

**FIGURE 2:**
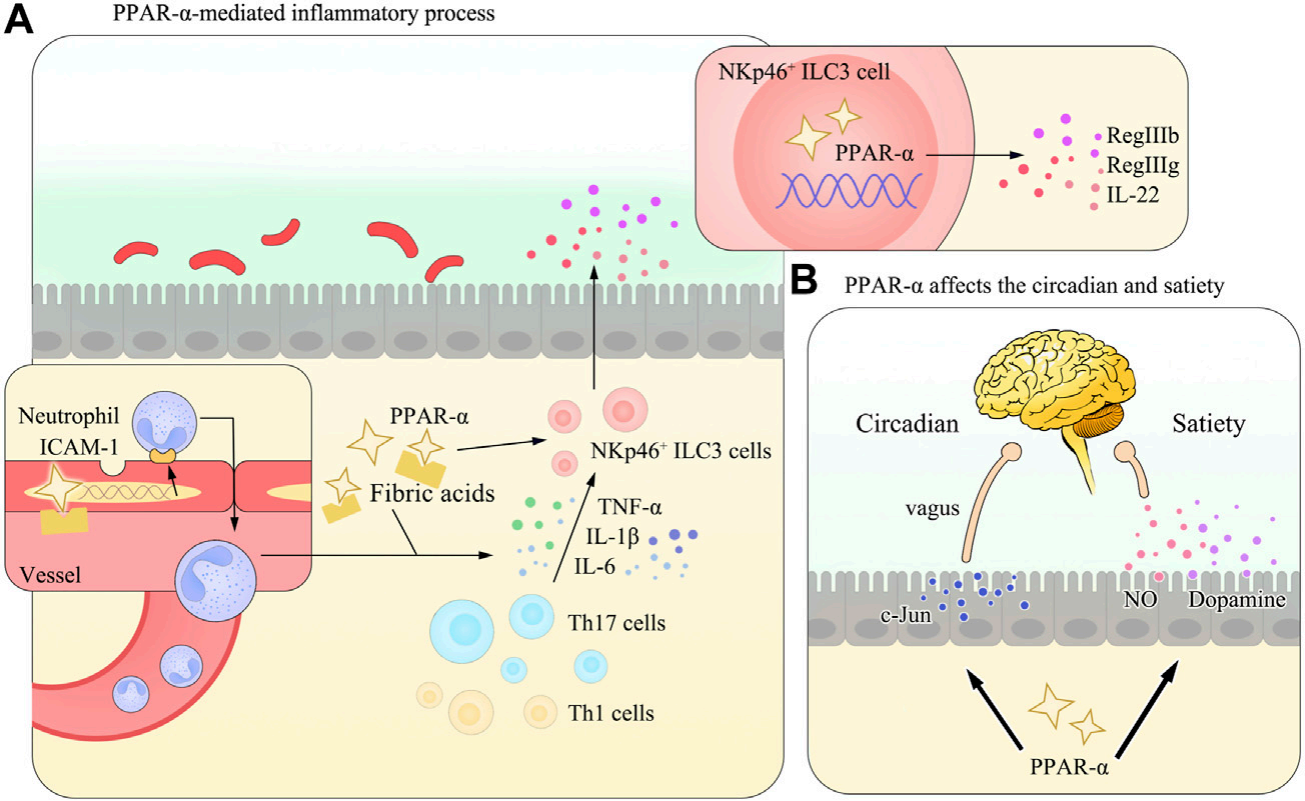
Diagram of the PPAR-α–mediated inflammatory process and the regulation of circadian rhythms or circadian clock and satiety in the gut. **(A)** In many gastrointestinal diseases, PPAR-α is activated and initiates the expression of multiple anti-inflammation mediators, including ICAM-1 in vascular epithelial cells and IL-22 in NKp46^+^ ILC3 cells. These effects help reverse the imbalance of the T-cell number and maintain the homeostasis of the GIT. **(B)** Apart from its role in regulating the inflammatory process, PPAR-α could also affect the circadian rhythms or circadian clock (*via* the regulation of the c-Jun expression) and satiety (by controlling the secretion of NO, as mentioned in the main body of the review, and Figure 3 and Table 3 are referred for detailed information) in the body. Studies have demonstrated its close relation with NO and dopamine, while the detailed mechanism remains to be elucidated.

Nitric oxide (NO) is one of the major biomarkers of GIT inflammation mainly synthesized by the inducible nitric oxide synthase (iNOS) enzyme in serum and affected tissues. It can exacerbate GIT inflammation and is elevated in times of colitis (Kamalian et al., 2020). Meanwhile, it also has close interplays with microbial components and liver metabolism (Yaguchi and Yaguchi, 2019). Studies using leukotriene B4, a PPAR-α agonist, have found naturally occurring PPAR agonists can inhibit the iNOS enzyme pathway. They further proposed the possibility of this modulation by the stress protein heme oxygenase 1, although the exact mechanisms wait for more investigations (Colville-Nash et al., 1998). Concomitantly, Fu et al. provided evidence for this correlation in OEA which was also recognized as a potential regulation of satiety (Fu et al., 2003; Sihag and Jones, 2018) (Refer to Figure 2 for vivid understanding).

### Circadian Rhythm Regulation in the Gastrointestinal Tract and its Interplays With Peroxisome Proliferator–Activated Receptor-α

**FIGURE 3:**
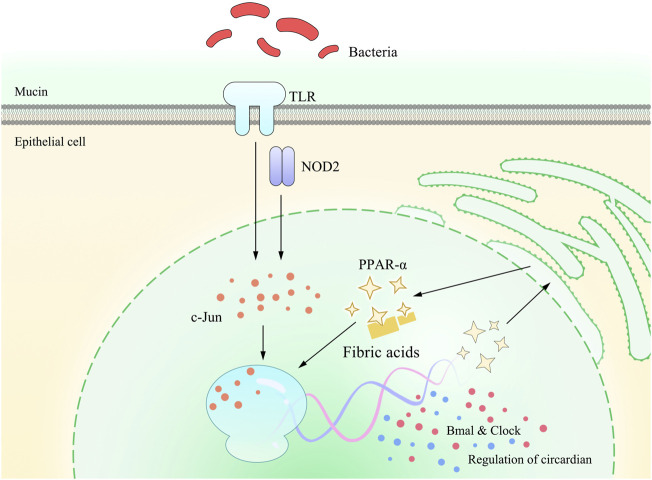
Pattern diagram of PPAR-α involved in TLRs and NOD2 on gastrointestinal circadian rhythms or circadian clock. Studies have found that gastrointestinal circadian rhythms or circadian clock is affected by the gut flora, which is mainly sensed *via* TLRs and NOD2. This stimulation is further detected by the c-Jun N-terminal kinase (JNK) and binds to the enhancer heptamer motif, resulting in the activation of PPAR-α and, in turn, activating the transcription of Bmal and Clock, which exert a direct impact on the regulation of gastrointestinal circadian rhythms or circadian clock.

Studies have found that circadian rhythms or circadian clock regulation is achieved *via* the expression of key genes and downstream pathways, as shown in Table 4. These genes take control of a broad range of physiological activities and share close interactions. One typical role of PPAR-α in this process lies in its activation of the clock gene *via* RORα and subsequent influence on the expression of E4BP4. However, as far as we are concerned, the current analysis focused on the roles of PPAR-α in the CVS, and more investigations focusing on its roles in GIT are recommended. Refer to Figure 3 for photographic illustration.

**TABLE 4:** Relative molecules involved in circadian rhythm regulation.

Abbreviation	Full name	Functions	References
TLR1-5,9	Toll-like receptor 1-5,9	Sense pathogen-associated molecular patterns	Lim and Staudt (2013)
NOD2	Nucleotide-binding oligomerization domain 2 (NOD2)	Senses bacterial peptidoglycan (PGN)–conserved motifs in cytosol	Okamura-Oho (2004)
RORE	Retinoic acid-related orphan receptor response elements	Regulating the expression of genes, including BMAL1 and CLOCK	Okamura-Oho (2004)
c-Jun	c-Jun	Binds to the enhancer heptamer motif and increased steroidogenic gene expression upon cAMP signaling pathway stimulation	Zhang et al. (2019)
RevErb	Reverb	Transcriptional repressor coordinating circadian rhythms or circadian clock rhythm and metabolic pathways in a heme-dependent manner	Prabhat et al. (2020)
RORα	Retinoid-related orphan receptor alpha (RORalpha)	Ligand-activated transcription factor involved in numerous biological processes	Liu et al. (2007)
Bmal	Brain and muscle ARNT-like1	Transcriptional activator which forms a core component of the circadian rhythms or circadian clock	Tognini et al. (2017)
Clock	Circadian rhythms or circadian clock locomotor output cycles kaput	Transcriptional activator which forms a core component of the circadian rhythms or circadian clock	Voigt et al. (2016)
DBP1	Dibutyl phthalate	Transcriptional activator recognizes and binds to the sequence 5′-RTTAYGTAAY-3′	Yu et al. (2019)
E4BP4	E4 promoter-binding protein 4 (E4BP4)	A transcriptional regulator that recognizes and binds to the sequence 5′-[GA]TTA[CT]GTAA[CT]-3′	Agarwal et al. (2018)
Cyp11a1	Cytochrome P450, family 11, subfamily A, polypeptide 1	Encoding a critical enzyme for steroid biosynthesis	Shih et al. (2008)

## Pharmacological Perspective of Peroxisome Proliferator–Activated Receptor-α

Numerous investigations have been put into the analysis of PPAR-α due to its wide distribution and multiple functions in a variety of tissues and cells (Berger and Moller, 2002). Long-chain fatty acids and their derivatives proved to be the main sources of natural PPAR-α agonists, while synthetic ones also play important roles in these studies (Kliewer et al., 1997). Although some agonists of PPAR-α have been used for treating different diseases, many of them are still in the experimental stage (Feng et al., 2016). Most clinical trials focused on their usage in metabolic diseases, especially those symptoms in the liver, while others concerning the gastrointestinal tract are relatively less (Petrosino et al., 2010). Also, considering the difference of distribution in humans and mice, more clinical trials are required in order to fully elucidate the mechanisms. Moreover, many exogenous nutrients and endogenous metabolites serve as ligands for PPAR-α while their functions and related dosage vary a lot. This increases the difficulty in developing clinical uses and requires further elucidation (O'Sullivan, 2016). However, we considered it worth the time and effort due to its potential usage in treating GIT diseases and decreasing the number of IBD patients all over the world.

## Conclusion

PPAR-α has been recognized as an important regulator in the cardiovascular system and lipid metabolism. In addition, it also exerts substantial impacts on the GIT functions both physiologically and pathologically. Other than the well-known abilities to regulate lipid metabolism, PPAR-α mediates the process of inflammation *via* the regulation of cytokine secretion and activation of inflammatory pathways. Furthermore, the target genes of PPAR-α include those controlling gut circadian rhythms and the synthesis of NO, which could form an integrated regulatory network of GI functions. Meanwhile, many endogenous and exogenous food metabolites serve as agonists of PPAR-α, and their use in the treatment of GIT diseases is expected to shed light for a bright future.

## Glossary

ABCA1: ATP-binding cassette transporter A1: ATP-binding cassette, sub-family A, member 1
ABCA1: ATP-binding cassette transporter A1: ATP-binding cassette, sub-family A, member 1
ACF: aberrant crypt foci
ANGPTL4, a circulating lipoprotein lipase: angiopoietin-like 4
Cd36: fatty acid translocase
CD36: cluster of differentiation 36
CGX: celiac superior mesenteric ganglionectomy
Chst4: carbohydrate (chondroitin 6/keratan) sulfotransferase 4
CNS: central nervous system
COX-2: cyclooxygenase-2
CPT 1A1: cytochrome P450 1A1
CVS: cardiovascular system
Cypt4a10: cytochrome P450, family 4, subfamily a, polypeptide 10
Dgat1: diacylglycerol acyltransferase 1
DSS: dextran sodium sulfate
Dtd Slc26a2: solute carrier family 26 (sulfate transporter), member 2
ERS: endoplasmic reticulum stress
F19: paracasei ssp paracasei F19
FAT: fatty acid translocase
FATP: fatty acid transport protein
Fatp4: fatty acid transport protein 4
GCs: glucocorticoids
GFT505: elafibranor
GIT: gastrointestinal tract
IBD: intestinal bowel diseases
IFABPs: intestinal fatty acid–binding proteins
IL-1b: interleukin-1b
iNOS: inducible nitric oxide synthase
IP: intraperitoneal
LBD: ligand-binding domain
LCFAs: long-chain fatty acids
mAspAT: mitochondrial aspartate aminotransferase
Mgst1: microsomal glutathione S-transferase 1
Mttp: microsomal triglyceride transfer protein
Mφ: macrophages
NO: nitric oxide
OEA: oleoylethanolamide
PEA: palmitoylethanolamine
PPAR: peroxisome proliferator–activated receptor
PPAR-αKO: PPAR-α-knockout mice
RXR; NR2B: retinoid X receptor
RYGB: Roux-en-Y gastric bypass
SAO: splanchnic artery occlusion
SDA: subdiaphragmatic vagal deafferentation
Sert Slc6a4: solute carrier family 6 (neurotransmitter transporter, serotonin), member 4
SIV: simian immunodeficiency virus
Slc25a36: solute carrier family 25, member 36
Smct1 Slc5a8: solute carrier family 5 (iodide transporter), member 8
TNF-a: tumor necrosis factor-alpha
Wy-14643: pirinixic acid
