# Corrigendum: Peroxisome proliferator–activated receptor-α: A pivotal regulator of the gastrointestinal tract

**DOI:** 10.3389/fmolb.2022.1063551

**Published:** 2022-10-21

**Authors:** Yue-Xin Guo, Bo-Ya Wang, Han Gao, Rong-Xuan Hua, Lei Gao, Cheng-Wei He, Ying Wang, Jing-Dong Xu

**Affiliations:** ^1^ Department of Oral Medicine, School of Basic Medical Sciences, Capital Medical University, Beijing, China; ^2^ Eight Program of Clinical Medicine, Peking University Health Science Center, Beijing, China; ^3^ Department of Physiology and Pathophysiology, School of Basic Medical Sciences, Capital Medical University, Beijing, China; ^4^ Clinical Medicine of “5+3” Program, School of Basic Medical Sciences, Capital Medical University, Beijing, China; ^5^ Department of Biomedical Informatics, Faculty of Biomedical Engineering, Capital Medical University, Beijing, China; ^6^ Department of Dermatology, Tongren Hospital, Capital Medical University, Beijing, China

**Keywords:** gastrointestinal diseases,, metabolism, transcription, disorder, peroxisome proliferator-activate receptor gamma (PPARγ)

In the published article, there were errors in the following affiliations.

Instead of “[1] Department of Oral Medicine, Basic Medical College, Capital Medical University, Beijing, China”, it should be “Department of Oral Medicine, School of Basic Medical Sciences, Capital Medical University, Beijing, China”.

Instead of “[3] Department of Physiology and Pathophysiology, Basic Medical College, Capital Medical University, Beijing, China”, it should be “Department of Physiology and Pathophysiology, School of Basic Medical Sciences, Capital Medical University, Beijing, China”.

Instead of “[4] Clinical Medicine of “5+3” Program, Basic Medical College, Capital Medical University, Beijing, China”, it should be “Clinical Medicine of “5+3” Program, School of Basic Medical Sciences, Capital Medical University, Beijing, China”.

The authors apologize for this error and state that this does not change the scientific conclusions of the article in any way. The original article has been updated.

